# Systemic immune remodeling following curative (R0) resection of colorectal liver metastases

**DOI:** 10.3389/fimmu.2026.1843400

**Published:** 2026-07-09

**Authors:** Shuo Ren, Migmar Tsamchoe, Stephanie K. Petrillo, Oran Zlotnik, Jessica Bloom, Anastasia Tsatoumas, Vered Domankevich, Anthoula Lazaris, Peter Metrakos

**Affiliations:** 1 Cancer Research Program, Research Institute of the McGill University Health Centre (RI-MUHC), Montreal, QC, Canada; 2 Department of Anatomy and Cell Biology, McGill University, Montreal, QC, Canada; 3 Alpha Tau Medical Ltd, Jerusalem, Israel; 4 Department of Surgery, McGill University, Montreal, QC, Canada

**Keywords:** CD4+ T cells, colorectal cancer liver metastases, immunotherapy, myeloid-derived suppressor cells, R0 resection, S100A9, systemic immunity

## Abstract

**Background:**

Colorectal cancer liver metastases (CRLM) are a key driver of systemic immunosuppression and a determinant of poor prognosis. While surgical resection remains the mainstay of treatment, the dynamics of the immune landscape post-resection remain insufficiently characterized. This study aims to delineate immune reprogramming following liver resection in CRLM patients, offering insights into potential therapeutic strategies.

**Methods:**

Peripheral blood samples were collected from CRLM patients before and after liver resection. Peripheral blood mononuclear cells were analyzed using multiparameter flow cytometry with adaptive and innate immunity panels, processed with FlowJo and FlowAI. Tumor immune microenvironment (TIME) was assessed by H&E, immunohistochemistry, and immunofluorescence.

**Results:**

Postoperative analysis revealed remodeling of T cell composition, with a significant increased proportion of CD4+ T cells among circulating CD3+ T cells, including the CD28+ CD4+ subset, while regulatory T cells and T follicular helper cells remained unchanged. Overall proportion of CD8+ T cells among circulating CD3+ T cells was reduced. Among immune checkpoint-associated populations, the percentage of TIM3+ CD4+ T cells decreased significantly, whereas PD-1+ CD4+ T cells and exhausted PD-1+ TIM3+ double-positive T-cell subsets showed modest downward trends. Innate immune populations remained largely unchanged. Patients who experienced recurrence had higher postoperative proportion of S100A9+ monocytic myeloid-derived suppressor cells (M-MDSCs). Exploratory analyses further suggested that KRAS-mutated tumors may be associated with distinct postoperative immune profiles.

**Conclusions:**

Liver metastasis resection is associated with CD4+ T cell-dominant systemic immune remodeling and changes in exhaustion-associated markers, hypothetically suggesting a potential postoperative window for future immunotherapeutic interventions. Although highly speculative and requiring further functional validation, these findings suggest that the altered CD4+ T cell landscape warrants further investigation regarding its potential relevance to adoptive cellular therapies or immune checkpoint inhibition.

## 1 Introduction

Colorectal cancer (CRC) ranks as the third most common cancer worldwide, with a global incidence exceeding 1.9 million cases and a mortality toll of 903, 859 in 2022 (1). Metastatic CRC (mCRC) represents a complex and multifaceted disease driven by diverse factors, continues to have a devastating prognosis with a survival rate of less than 15% (2). Over the past decades, treatment strategies for most colorectal liver metastases (CRLM) have largely relied on chemotherapy. The addition of targeted therapies against vascular endothelial growth factor receptor (VEGFR) and epidermal growth factor receptor (EGFR) signaling pathways has only led to improved outcomes in specific patient subsets (3, 4). Indeed, neoadjuvant therapy combined with surgical R0 resection remains the cornerstone of potentially curative treatment for CRLM. However, relapse occurs in up to 55% to 80% of patients following metastatic resection (5). In recent years, immune checkpoint inhibitors targeting programmed cell death protein 1 (PD-1) has demonstrated clinical benefits, but their efficacy is limited to a small subset of patients which are MSI (microsatellite instability) (6, 7). Given the well-established association between the progression of CRLM and systemic immunity (8), gaining a deeper understanding of the systemic immune landscape in CRLM is essential for developing more effective therapeutic strategies.

The liver is essential for maintaining immune tolerance, especially in autoimmune diseases, organ transplantation, and cancer (9–11). Mechanisms underlying hepatic immune tolerance involve interactions between liver-resident antigen-presenting cells—like Kupffer cells, dendritic cells, and sinusoidal endothelial cells—and circulating lymphocytes (12). Furthermore, the liver is the tolerance-inducing organ for transplantation, allowing some long-term recipients to discontinue immunosuppressive therapy without experiencing graft rejection (13). In cancer, liver metastasis is associated with reduced immunotherapy response and shorter progression-free survival compared to patients without liver metastasis (14). Notably, preclinical mouse models show that (11) liver metastases hinder immunotherapy responses in subcutaneous and hepatic tumors. However, liver-directed radiotherapy can restore immunotherapy effectiveness against extrahepatic disease and enhance systemic antitumor immunity.

CD4+ T cells play a pivotal role in coordinating both innate and adaptive immune responses (15). During the initiation of anti-tumor immune responses, CD4+ T cells activate APCs (Antigen-Presenting Cells), facilitating the effective priming of cytotoxic T lymphocytes and supporting their differentiation into long-lasting memory cells (16, 17). Moreover, CD4+ T cells secrete tumor necrosis factor (TNF) and IFNγ, which induce macrophage polarization into an M1-like tumoricidal phenotype capable of eliminating tumor cells through the production of nitric oxide (18). Interestingly, in addition to their helper functions in anti-cancer immunity, evidence in humans has shown that a subset of CD4+ T cells can directly eradicate cancer cells, similarly to classical CD8+ T cells (19). However, CD4+ T cells may also facilitate tumor initiation and progression through regulatory T cell-mediated immunosuppressive functions. Consistent with this concept, previous studies have reported an increased frequency of circulating regulatory T cells during the adenoma-to-carcinoma transition (20).

In both humans and mice, myeloid-derived suppressor cells (MDSCs) in the innate immune system are broadly categorized into two primary subsets: granulocytic MDSCs (G-MDSCs) and monocytic MDSCs (M-MDSCs) (21). These subsets are distinguished by their origin from the granulocytic or monocytic branches of the myeloid lineage, respectively. M-MDSCs accumulation plays a critical role in creating an immunosuppressive TME in the liver (22). By producing CCL7 that binds CCR2 on micro-metastatic cells, MDSCs activate dormant cells via the JAK/STAT3 pathway and promote CRLM progression (23). In addition, MDSCs not only aid in evading immune surveillance but also support cancer cell survival through non-immune mechanisms, such as stimulating angiogenesis, driving tissue remodeling, and facilitating metastasis (24, 25). However, their heterogeneity poses a significant challenge for effectively targeting and inhibiting their functions (26). Therefore, investigating the levels and dynamics of circulating MDSCs in CRLM patients is crucial for developing targeted therapeutic strategies.

Importantly, peripheral immune signatures have been shown to reflect tumor burden and metastatic patterns. In gastrointestinal malignancies, alterations in circulating myeloid subsets and T-cell exhaustion phenotypes have been associated with advanced disease and liver metastasis (11, 27). Recent integrative multi-omic analyses further highlight that peripheral immune states can recapitulate intratumoral transcriptional programs, underscoring the relevance of blood-based immune profiling as a surrogate for tissue immunology (28).

In this study, we aim to characterize the changes and patterns of the systemic immune landscape before and after liver resection and explore the correlation between key immune features associated with clinical outcomes and disease progression.

## 2 Materials and methods

### 2.1 Human clinical sample collection

This study was conducted in compliance with the guidelines of the Institutional Review Board (IRB) at McGill University Health Centre (MUHC) (protocol # SDR-11-066). Inclusion Criteria: Patients were eligible for inclusion if they had a confirmed diagnosis of resectable CRLM with curative intent and showed no evidence of distant metastases beyond the liver. The majority of patients had undergone resection of their primary tumor prior to liver surgery, allowing for an accurate assessment of baseline immune status confined to liver-only disease. Written informed consent was obtained from all participants prior to enrollment. Blood samples were obtained from the MUHC Liver Disease Biobank and were collected from patients undergoing lobectomy or partial hepatectomy at the Royal Victoria Hospital in Montreal, QC, Canada. Surgical resections were performed between December 2022 and May 2024. Clinical data were extracted from hospital databases and medical records. Patient data were collected and analyzed up to 25th June 2024, which served as the data cutoff date for this study.

A total of 26 patients were initially eligible for our study. Of these, nine were subsequently excluded based on the following criteria (**Supplementary Figure 1**): one due to a non-R0 resection, one because no liver metastases were detected during intraoperative ultrasound, and two following a pathological diagnosis of metastatic small bowel tumor. Additionally, five patients who experienced very early postoperative recurrence, confirmed within two months through elevated carcinoembryonic antigen (CEA) levels, contrast-enhanced CT or MRI findings, and physical examination, were excluded from the study. These patients were excluded because very early recurrence likely reflected the presence of occult residual or rapidly progressive micro metastatic disease at the time of surgery, which could substantially confound the assessment of postoperative systemic immune remodeling following curative-intent R0 resection. Therefore, the present analysis was designed to evaluate perioperative immune changes in patients who achieved an initial disease-free postoperative course with minimum two months. Consequently, blood samples from the remaining 17 patients with CRLM were collected in the operating room (OR) prior to surgical incision and postoperatively during the disease-free follow-up period, which ranged from 3.0 to 16.9 weeks after liver resection. Detailed preoperative and postoperative sampling timelines are provided in **Supplementary Table 1**.

### 2.2 Flow cytometry

In summary, 4–6 ml whole-blood samples were collected and stored in EDTA tubes (BD Vacutainer Plastic Blood Collection Tubes with K2EDTA: Hemogard Closure 6mL; Fischer). Peripheral blood mononuclear cells (PBMCs) were isolated from the blood using the EasySep Direct Human PBMC Isolation Kit (Stem Cell; Cat #19654) and EasySep magnet (Stem Cell; Cat #18001), following the manufacturer’s protocol. To detect and exclude dead cells, eFluor780 viability dye (ThermoFisher Scientific; Cat #65-0865-14) was added to each sample and incubated on ice, covered with aluminum foil, for 20 minutes. After washing with stain buffer (BD Biosciences; Cat #554656), the cells were incubated with an extracellular antibody cocktail, as listed below, for 30 minutes. The cells were then washed with 1 ml stain buffer and resuspended in 1 ml freshly prepared Fix/Perm buffer (BD Biosciences; Cat #562574) for 40–50 minutes. After washing with Perm/Wash buffer (BD Biosciences; Cat #562574), the cells were subjected to staining with intracellular antibodies, as listed below. Compensation controls using beads were conducted to ensure consistent cytometer settings. Additionally, Fluorescence Minus One (FMO) controls were performed for the markers to establish negative gating.

Two panels were developed (each utilizing 1 million PBMCs): an innate immunity panel, designed primarily to characterize myeloid-derived cell population, and an adaptive immunity panel, aimed at examining T cell expression and immune checkpoint markers to explore the systemic immunosuppressive milieu associated with liver metastasis. The following antibodies were used: Innate immunity panel: CD45 (Clone HI30, BUV395, BD Biosciences; Cat #563792), CD11b (Clone M1/70, BV650, BioLegend; Cat #101259), CD14 (Clone MφP9, PerCP, BD Biosciences; Cat #340585), CD15 (Clone HI98, PE, BD Biosciences; Cat #555402), HLA-DR (Clone L243, PE-Cy7, BioLegend; Cat #307616), CD20 (Clone 2H7, BUV737, BD Biosciences; Cat #612848), CD3 (Clone UCHT1, BV510, BD Biosciences; Cat #563109), MRP14 (Clone MRP 1H9, APC, BioLegend; Cat #350708). Adaptive immunity panel: CD45 (same as innate immune panel), CD4 (RPA-P4, FITC, BD Biosciences; Cat #555346), CD8 (Clone RPA-T8, APC, BD Biosciences; Cat # 555369), CD279 (PD-1) (Clone EH12.1, BV711, BD Biosciences; Cat #564017), CD366 (Clone 344823, BV650, BD Biosciences; Cat #747960), CD178 (Fas Ligand) (Clone NOK-1, BV605, BD Biosciences; Cat #744099), CD185 (Clone RF8B2, BB700, BD Biosciences; Cat #566469), CD25 (Clone M-A251, PE-Cy7, BD Biosciences; Cat 61405), CD28 (Clone CD28.2, PE-CF594, BD Biosciences; Cat #562296), LAG-3 (Clone T47-530, BD Biosciences; Cat #565616), CD138 (Clone MI15, BUV737, BD Biosciences; Cat #612834), CD3 (same as innate immune panel), FoxP3 (Clone 259D/C7, R718, BD Biosciences; Cat #566935). Representative flow cytometry gating is shown by (**Supplementary Figure 2**, **S4**).

### 2.3 Flow cytometry data analysis

FlowJo_v10.10.0 was utilized for downstream analysis, while Flow AI was employed for quality control of all samples. This tool effectively removes unwanted events from flow cytometry data, ensuring high-quality analysis (29). Only cell populations in which the proportion of “Good Events” exceeded 70%, as determined by Flow AI filtering, were included in the analysis. It should be noted that absolute immune cell counts were not available in this study.

### 2.4 Hematoxylin and eosin staining

We performed staining for formalin-fixed paraffin-embedded (FFPE) specimens as described in previous publications (30). Briefly, resected CRCLM patient samples were formalin-fixed and paraffin-embedded (FFPE), then sectioned at 4 μm for histopathological evaluation. Following fixation in paraformaldehyde and embedding, sections were placed on charged slides. CRLM sections were deparaffinized in xylene and rehydrated through a graded ethanol series. Sections were rinsed in distilled water and stained with hematoxylin, followed by a brief incubation in a bluing reagent. After rinsing, slides were counterstained with eosin, dehydrated through graded ethanol, cleared in xylene, and mounted with Permount™ mounting medium. Stain sections were allowed to dry completely before imaging.

### 2.5 Immunohistochemistry

Immunohistochemical analyses were performed on FFPE CRLM tumor sections following standard protocols (31). Antigen retrieval was conducted according to the manufacturer’s instructions. Sections were incubated overnight at 4 °C with primary antibodies against TIM3 (Abcam, ab241332, 1:1000), CD4 (Abcam, ab133616, 1:1200), CTLA4 (Abcam, ab237712, 1:250). Detection was performed using appropriate HRP-conjugated secondary antibodies (Dako, K4003). Stained slides were scanned at 20× magnification using the Aperio AT Turbo system. Quantitative analyses of marker-positive cells were performed in defined regions of interest.

### 2.6 Immunofluorescence

Paraffin-embedded tissue sections were deparaffinized and rehydrated using standard procedures. Antigen retrieval was carried out by heat treatment in Tris/EDTA buffer (10 mM, pH 9.0). Tissue permeabilization was performed with PBS buffer (PBS containing 0.5% Triton X-100), followed by blocking with 5% goat serum in PBST for 1 hour at room temperature. Sections were incubated overnight at 4 °C with FITC-conjugated anti-CD68 antibody (ThermoFisher Scientific, MA1-82715, 1:20) and anti-TIM3 primary antibody (Abcam, ab241332, 1:300). After washing with PBST, slides were incubated with Alexa Fluor™ 647-conjugated goat anti-rabbit IgG secondary antibody (Invitrogen, A27040) for 1 hour at room temperature. Nuclear counterstaining was subsequently performed using DAPI (Thermo Fisher Scientific, 62248, 1:1000) for 10 minutes. Finally, sections were mounted with Fluoromount (Invitrogen, 5018788), and fluorescence images were acquired using a Zeiss LSM780 laser scanning confocal microscope.

### 2.7 Statistical analysis

Statistical comparison among regions of interest were performed using GraphPad Prism software (version 9.5.1) and SPSS (version 27). Data were presented as mean ± SD or median with IQR, as appropriate. Statistical significance was assessed using suitable tests based on the data distribution. Comparison between two groups was conducted using a two-tailed t-test. Correlation analyses between clinical features and immune cell frequencies were evaluated using the Spearman rank correlation coefficient. The pairwise correlation matrix was visualized using a heatmap reflecting correlation strength. P value < 0.05 were considered statistically significant.

## 3 Results

### 3.1 Baseline demographics and clinical features

This study included 17 patients with CRLM, all of whom underwent liver resection and adjuvant therapy at the MUHC. The baseline characteristics of the patients, including age, sex, NGS status, and chemotherapy history etc., are summarized in (**Supplementary Table 2**). Most patients had undergone primary tumor resection prior to hepatic surgery. One patient underwent simultaneous resection of both the primary colorectal lesion and liver metastases, while another received a procedure aimed at anal preservation. Additionally, 58.8% of patients presented with KRAS mutation in the primary tumor. Neoadjuvant chemotherapy was administered to 64.7% of patients prior to resection of liver metastases. Of note, we also analyzed the baseline characteristics of the excluded patients, our analysis demonstrated no significant differences in all clinical parameters.

### 3.2 Increased frequency of CD4+ and decreased frequency of CD8+ T cells following liver resection in CRLM patients

**Figure 1:**
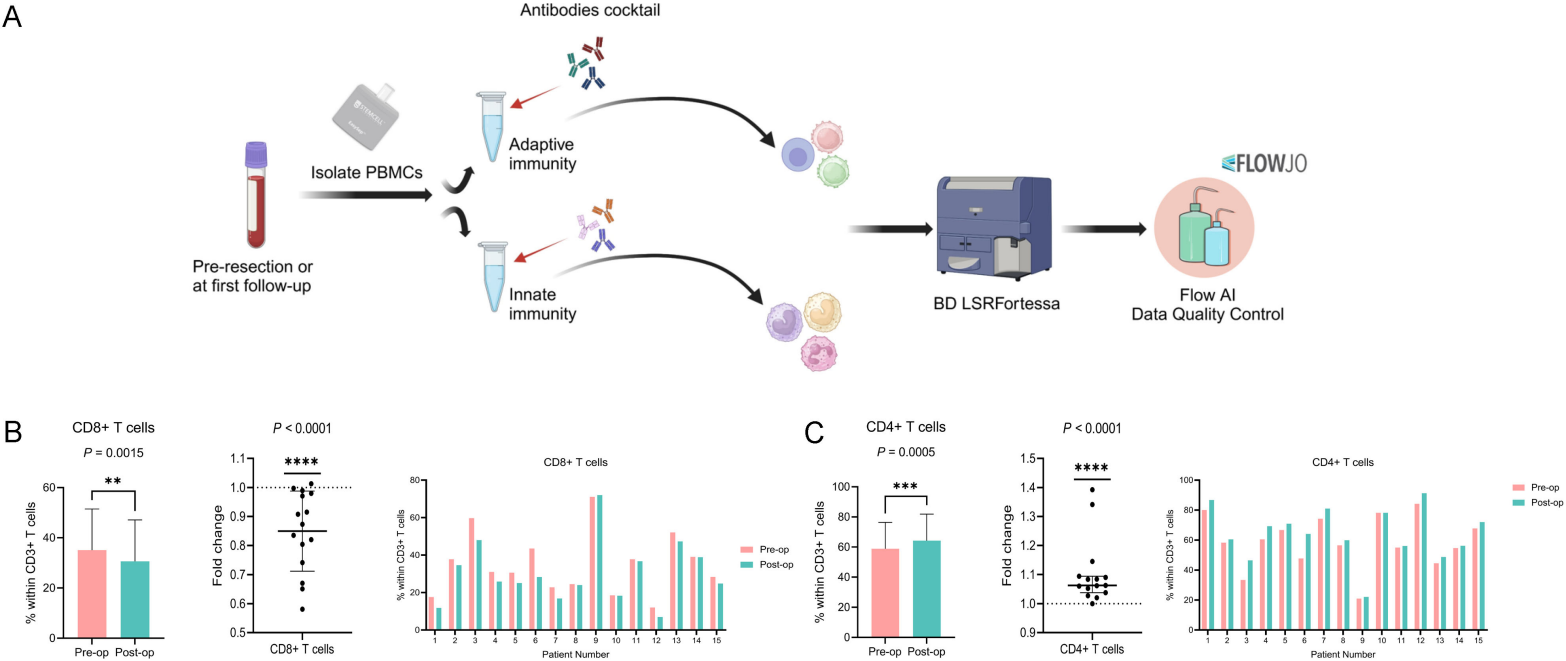
Dynamics of CD8+ and CD4+ lymphocytes following liver resection (n = 15). **(A)** Schematic of the experimental workflow. Created in BioRender. Gantz, A. (2026) https://BioRender.com/kftxjkf. **(B)** Analysis of circulating CD8+ T cells: comparison of pre- and post-operative percentages, fold changes (post-operative vs. pre-operative), and individual patient trajectories. Data are shown as mean ± standard deviation (SD) or median ± IQR, depending on the distribution. **(C)** Percentage, fold change (post-operative vs. pre-operative), and individual patient data for circulating CD4+ T cells. Data are shown as mean ± SD or median ± IQR, as appropriate. Error bars indicate SD or IQR. P < 0.05 was considered statistically significant. *P < 0.05; **P < 0.01; ***P < 0.001; ****P < 0.0001.

To investigate changes in immune populations, we established two panels: one focused on adaptive immunity and the other on innate immunity. T lymphocytes and their subpopulations were analyzed using the adaptive immunity panel. For each panel, 10–000 events were collected using the LSRFortessa™ cell analyzer. The Flow AI plugin in FlowJo software was then applied to filter and retained “Good Events” for subsets analysis (**Figure 1A**). Of the 17 patients initially included, 15 (n = 15) were analyzed in the adaptive panel, as two samples had less than 70% “Good Events”. The gating strategy is presented in **Supplementary Figure 2**.

We assessed the relative proportions of CD8+ and CD4+ T cells among CD3+ T cells isolated from peripheral blood, stratified by pre-operative and post-operative timepoints using column or individual plots. The frequency of CD8+ T cells was significantly decreased in the disease-free state compared to pre-operative controls (p = 0.0015), whereas the frequency of CD4+ T cells was significantly increased following the resection of liver metastases (p = 0.0005). A fold change analysis (post-operative/pre-operative) revealed values significantly different from a ratio of 1 for both CD8+ and CD4+ populations (p < 0.0001) (**Figures 1B, C**). Together, these results reflect a distinct postoperative remodeling of systemic T-cell composition.

### 3.3 Expression patterns of CD28 and CD178 on CD8+ and CD4+ T cells before and after liver resection

**Figure 2:**
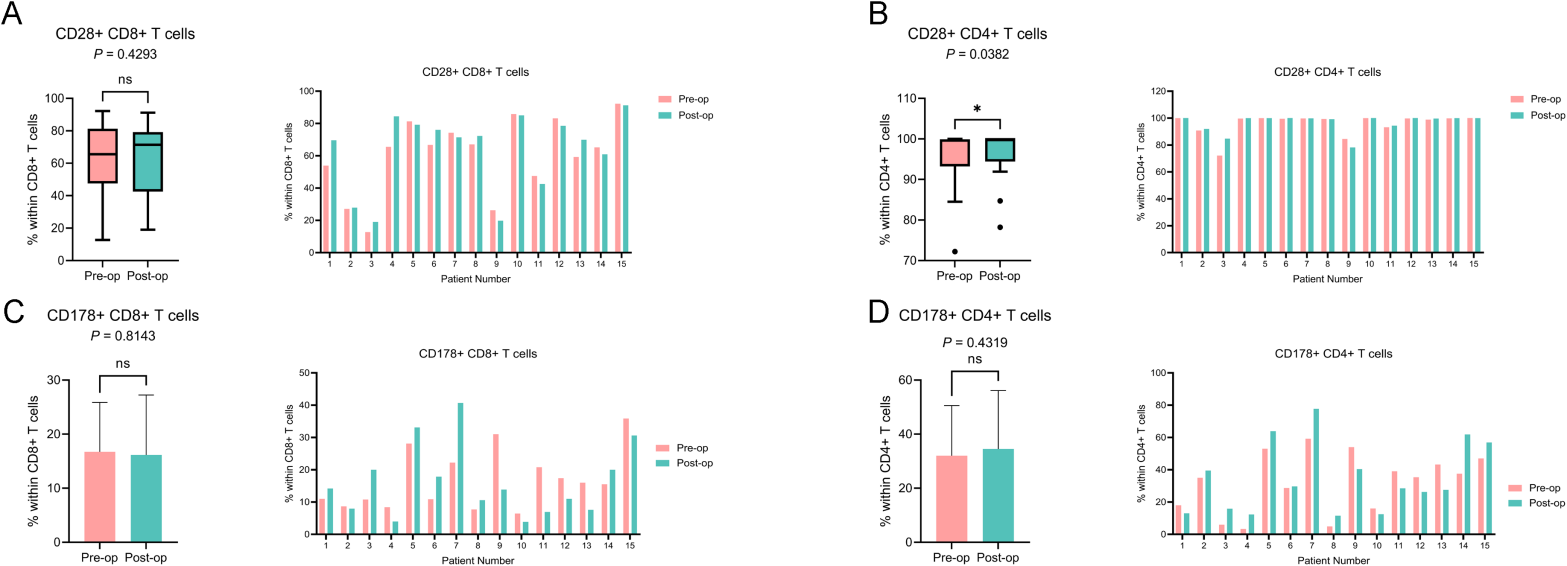
Expression levels of CD28 and CD178 (FasL) on T lymphocytes (n = 15). **(A, B)** Pre- and post-operative expression of CD28 on CD8+ and CD4+ T lymphocytes, with individual patient trajectories illustrating changes. Data are presented as median ± IQR. **(C, D)** Pre- and post-operative levels of CD178 (FasL) on CD8+ and CD4+ T lymphocytes, highlighting variability across patients. Data are shown as mean ± SD or median ± IQR, as appropriate. Error bars indicate SD or IQR. P < 0.05 was considered statistically significant. *P < 0.05. Statistical significance was assessed using paired t-tests. *P < 0.05.

Engagement of the CD28 receptor on T cells delivers an essential co-stimulatory signal that works in tandem with T cell receptor (TCR) activation to initiate naive T cell activation (32). To better understand the functional status of T cells in the context of CRLM, we assessed CD28+ expression on T lymphocytes. Our results demonstrated no significant change in the percentage of CD28+ CD8+ T cells following liver resection, with the mean values of 60.53% and 63.18% in the pre-operative and post-operative groups, respectively (**Figure 2A**). However, the percentage of CD28+ CD4+ T cells increased significantly after surgery (p = 0.0382) and the mean value elevated to 96.49% in post-operative group (**Figure 2B**). CD178 (FasL, CD95L, TNFSF6), a member of the tumor necrosis factor family of death factors, plays a role in the cytotoxicity of T and NK cells through Fas/FasL-mediated apoptosis (33). To investigate whether liver resection provided any advantage in the post-operative group compared to the pre-operative group, we analyzed CD178 expression. As shown in **Figures 2C, D**, no significant differences were observed in CD178 expression on both CD8+ and CD4+ T cells following resection.

### 3.4 Immune landscape of cell subsets and immune checkpoints before and after surgery

To further delineate the immune alterations associated with liver resection, we analyzed the frequencies of key immune cell subsets and surface indicators of T cell exhaustion. The Tfh subpopulation, characterized as CD3+CD4+CXCR5+ (**Supplementary Figure 2C**), displayed no significant differences pre- and post-operatively, with mean frequencies of 8.044% and 8.652%, respectively (p = 0.6953) (**Supplementary Figure 3A**). Similarly, Treg cells, identified as CD3+CD4+CD25+FoxP3+ (**Supplementary Figure 2C****),** showed consistent levels before and after surgery, with mean frequencies of 2.273% and 1.931% (p = 0.2545) (**Supplementary Figure 3B**). B cells (CD45+CD3-CD20+) also remained stable, with no significant changes observed between the pre- and post-operative groups (p = 0.8536) (**Supplementary Figures 3C**, **4B**).

**Figure 3:**
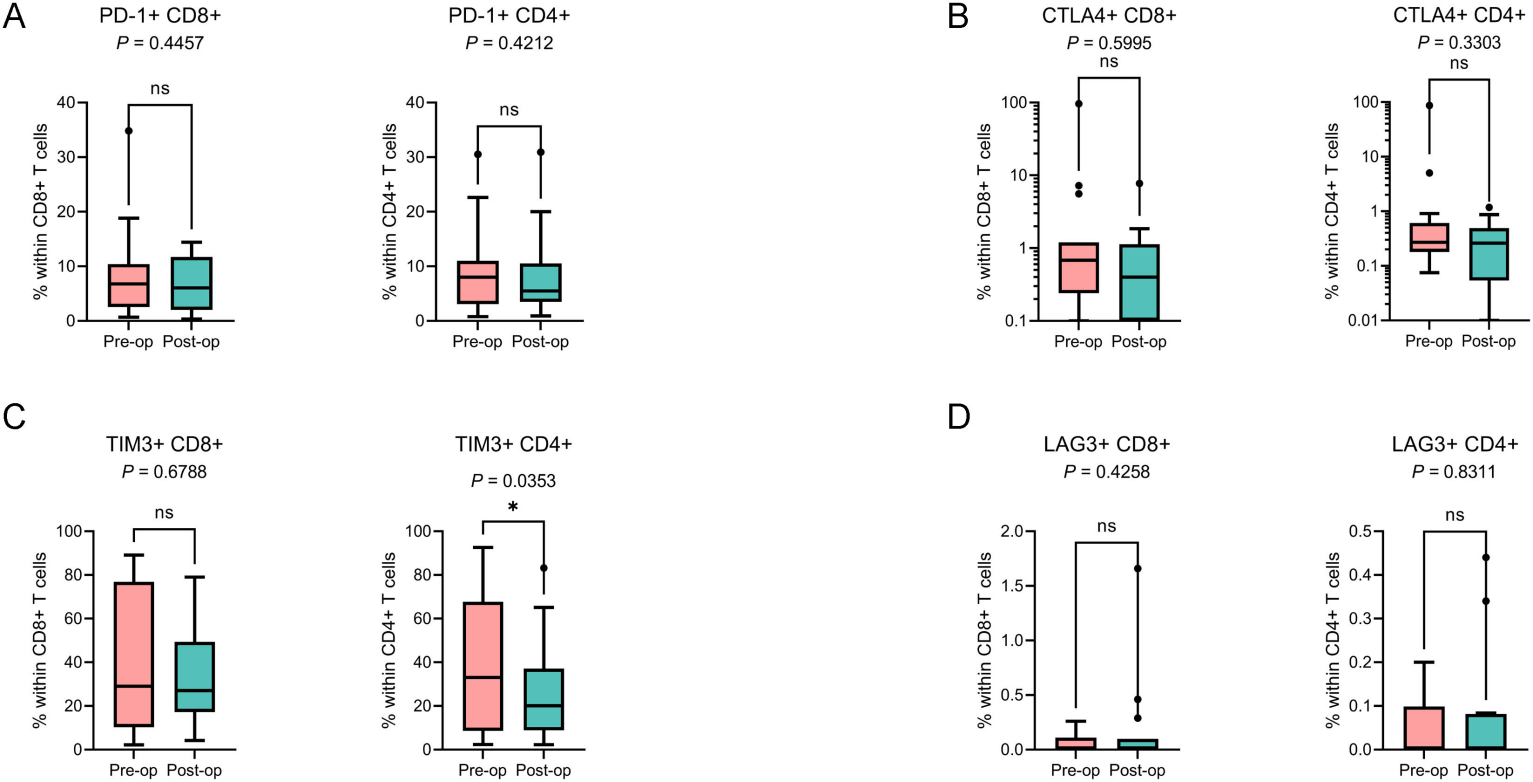
Expression of co-inhibitory markers on CD8+ and CD4+ T lymphocytes. **(A–D)** The frequency of immune checkpoint markers (PD-1, CTLA4, TIM3, and LAG3) on CD8+ and CD4+ T cells in peripheral blood was analyzed by flow cytometry in 15 patients with CRLMS before and after surgery. Data are presented as median ± IQR. Statistical comparisons were performed using paired t-tests, with significance defined as P < 0.05. *P < 0.05.

Furthermore, we examined four distinct coinhibitory markers-PD-1, CTLA4, TIM3 and LAG3-known to be critically associated with CD8+ and CD4+ T cells exhaustion in CRLM patients (34). Among these, the frequency of TIM3+ CD4+ T cells was significantly reduced post-surgery, decreasing from a mean of 39.98% pre-operatively to 25.70% post-operatively (p = 0.0353) (**Figure 3C**). By contrast, no significant changes were observed in the expression of PD-1, CTLA4, or LAG3 on either CD8+ or CD4+ T cells (**Figures 3A, B, D**). Additionally, a decreasing trend in the frequencyxof PD-1+ TIM3+ double-positive cells was observed in both CD4+ and CD8+ T-cell subsets after liver resection (**Supplementary Figure 5**).

In the innate immune panel, the frequencies of myeloid cells (CD11b+), monocytes, and M-MDSCs were unchanged following surgery (p > 0.05) (**Supplementary Figures 3D–F**, **4A**), indicating a broadly preserved innate immune profile. Overall, these results suggest a selective postoperative remodeling of T-cell exhaustion features, while innate immune populations remain largely unaffected.

To ensure that these selective postoperative T-cell remodeling patterns and checkpoint alterations were not confounded by the relatively broad blood collection window (3.0 to 16.9 weeks) or ongoing surgical wound healing kinetics, we performed a thorough validation analysis (**Supplementary Figure 6**). Correlation analyses demonstrated that postoperative sampling intervals were not significantly associated with the frequencies of key circulating immune populations, including CD4+ T cells, CD8+ T cells, CD28+ CD4+ T cells, TIM3+ CD4+ T cells, and S100A9+ M-MDSCs (**Supplementary Figure 6A**). Furthermore, when the cohort was stratified into early (≤ 5 weeks postoperatively) and late (> 5 weeks postoperatively) sampling groups, the characteristic trajectories observed in the overall cohort—specifically the significant postoperative increase in the proportion of CD4+ T cells, decrease in the proportion of CD8+ T cells, and reduction in the proportion of TIM3+ CD4+ T cells—remained evident in the early sampling group and showed similar trends in the late sampling group. In addition, no significant differences in postoperative levels of these key immune subsets were observed between the early and late sampling groups (**Supplementary Figures 6B–D**).

Collectively, these findings support that the observed systemic immune alterations are primarily associated with tumor mass removal rather than variations in postoperative recovery kinetics.

### 3.5 Impact of neoadjuvant chemotherapy on systemic immune cell expression in CRLM patients

**Figure 4:**
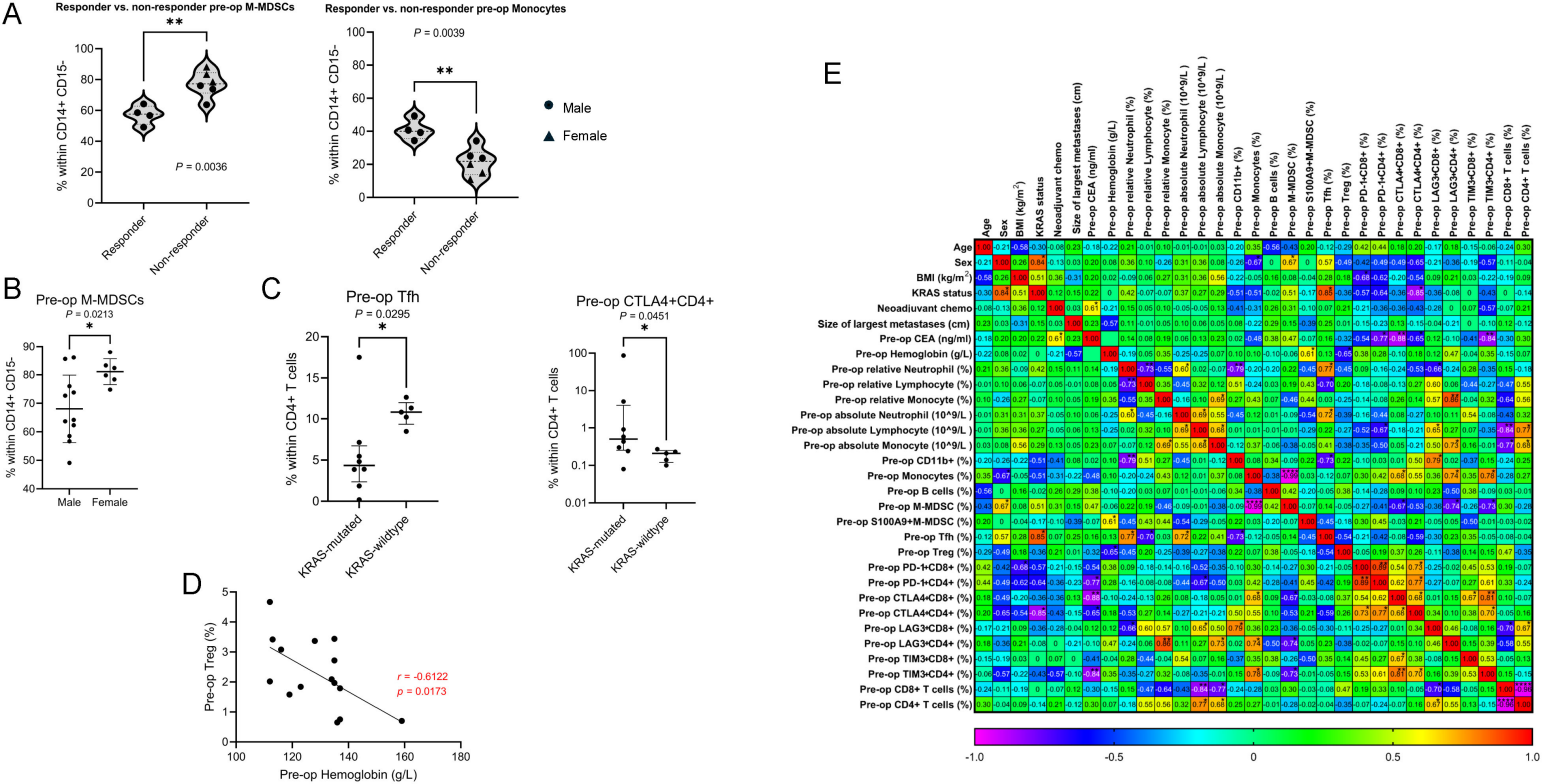
Myeloid cell alterations associated with neoadjuvant chemotherapy response and correlations between immune subsets and clinical parameters. **(A)** Comparison of preoperative M-MDSCs and Monocytes frequencies between chemotherapy responders and non-responders. **(B)** Sex-associated difference in preoperative M-MDSCs frequencies. **(C)** Comparison of preoperative Tfh and CTLA4+ CD4+ T-cell frequencies between KRAS-mutated and wild-type patients. **(D)** Correlation between preoperative Treg frequencies and hemoglobin levels. **(E)** Spearman correlation matrix integrating clinical features, preoperative complete blood count parameters, and preoperative flow cytometry data. The heatmap displays pairwise Spearman correlation coefficients (r), with color intensity reflecting the correlation strength.

Neoadjuvant chemotherapy is a cornerstone in the management of metastatic colorectal cancer. We hypothesized that such treatment might influence systemic immune profiles. To test this, we stratified the entire cohort according to whether patients had received neoadjuvant chemotherapy targeting liver metastases (**Supplementary Table 2**). All patients who underwent neoadjuvant therapy received standard 5-fluorouracil–based regimens (FOLFOX, CAPOX, or FOLFIRI). We then compared peripheral immune cell populations between the two groups at both the preoperative and postoperative time points. Stratification by neoadjuvant chemotherapy administration revealed no significant differences in either adaptive or innate immune compartments at both time points (p > 0.05; **Supplementary Table 3**). In contrast, patients who responded to therapy exhibited significantly lower preoperative levels of circulating M-MDSCs and higher levels of monocytes compared with non-responders (**Figure 4A**). These findings suggest that elevated preoperative M-MDSC levels may be associated with reduced treatment sensitivity.

It should be noted that neoadjuvant therapy for liver metastases was discontinued 4 weeks prior to sample collection and surgical resection.

### 3.6 Exploratory analysis of Immune cell-clinical parameter associations

**Figure 5:**
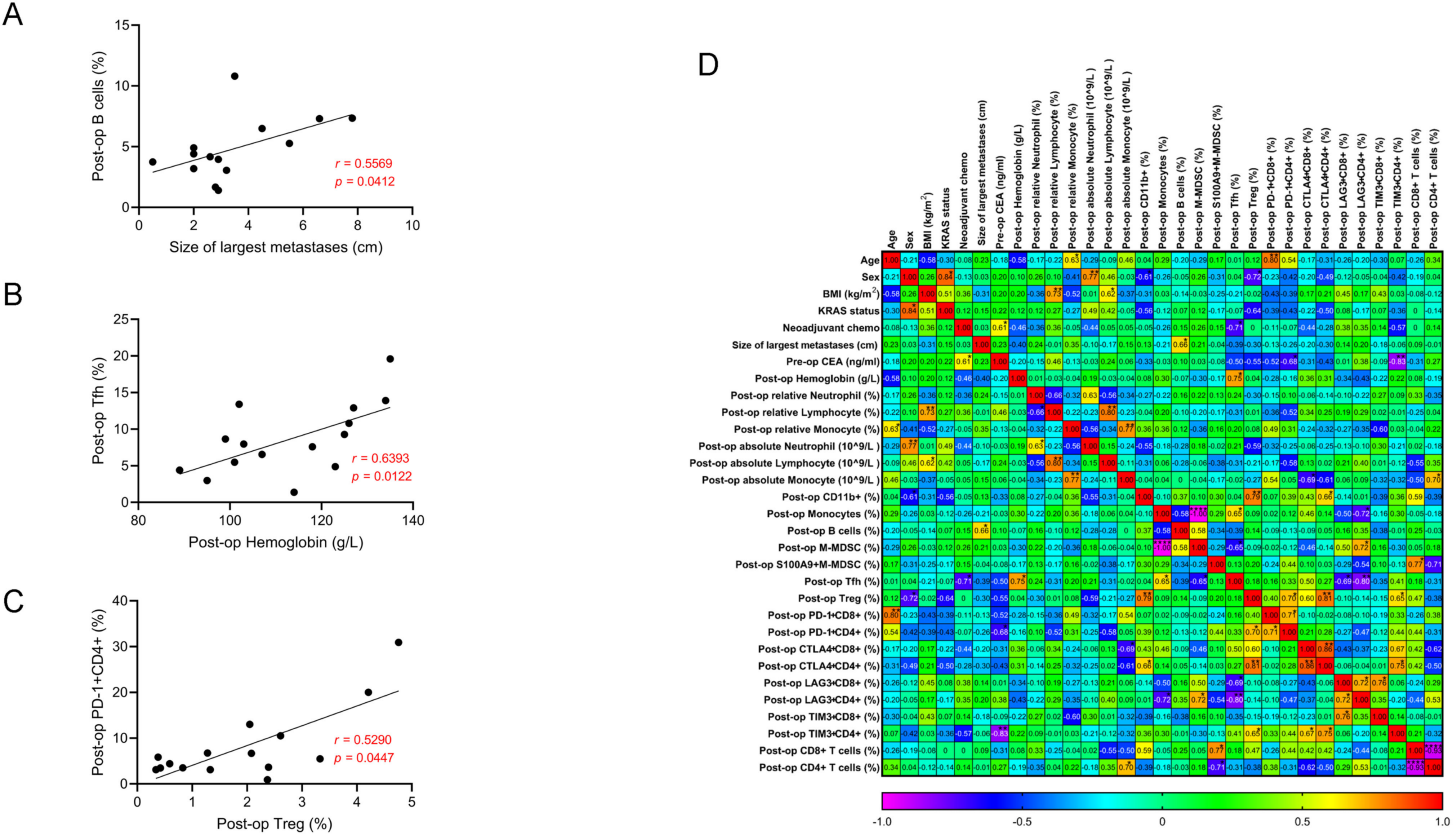
Correlations between immune subsets and clinical parameters. **(A)** Correlation between postoperative B cells frequencies and size of largest metastases. **(B)** Correlation between postoperative Tfh frequencies and hemoglobin levels. **(C)** Correlation between postoperative Treg and PD-1+ CD4+ frequencies. **(D)** Spearman correlation matrix integrating clinical features, postoperative complete blood count parameters, and postoperative flow cytometry data. The heatmap displays pairwise Spearman correlation coefficients (r), with color intensity reflecting the correlation strength.

To exploratorily explore potential associations between circulating immune cell subsets and clinical parameters, we performed Spearman’s correlation analyses across all measured variables, generating two comprehensive correlation matrices corresponding to preoperative and postoperative datasets (**Figures 4E**, **5D**). Variables exhibiting strong (|r| ≥ 0.6) and statistically significant correlations were marked with asterisks.

In the preoperative dataset, sex showed a significant positive correlation with circulating M-MDSC levels (r = 0.666, p < 0.05; **Figure 4E**). To further validate this finding, we compared preoperative M-MDSC frequencies between male and female patients, which demonstrated that female patients displayed significantly higher circulating M-MDSC levels before surgery (**Figure 4B**), suggesting a preoperative myeloid-derived immunosuppressive state in female patients. Interestingly the female data points were more consistent between patients than in the male cohort which demonstrated a lot of variability. Although the data set is small this significance warrants more investigation.

KRAS mutation status was significantly correlated with two adaptive immune subsets, showing a positive correlation with preoperative Tfh cells (r = 0.853, p < 0.05) and a negative correlation with preoperative CTLA4+CD4+ T cells (r = –0.853, p < 0.05; **Figure 4E**). Consistent with these correlations, group comparison demonstrated that KRAS-mutated patients had significantly lower preoperative Tfh frequencies and higher CTLA4+CD4+ T-cell percentages compared with the KRAS wild-type group (**Figure 4C**). Additionally, preoperative hemoglobin levels were inversely correlated with circulating Treg frequency (**Figure 4D**). Collectively, these findings suggest that KRAS-mutated patients exhibit an immune-exhausted and immunosuppressive phenotype, and that systemic hypoxia or anemia may contribute to elevated circulating Treg levels.

In the postoperative dataset (**Figure 5**), the maximum diameter of liver metastases was positively correlated with the postoperative proportion of circulating B cells (r = 0.557, p = 0.041; **Figure 5A**), suggesting that patients with larger tumor burden retain higher systemic B-cell levels after surgery. Postoperative hemoglobin levels were also positively correlated with circulating Tfh frequencies (r = 0.639, p = 0.012; **Figure 5B**). Moreover, postoperative PD1+CD4+ T cells were positively associated with postoperative Treg levels (r = 0.529, p = 0.045; **Figure 5C**).

Collectively, while these statistical associations point toward novel links between systemic immunity and clinical features, we acknowledge that these preliminary findings are restricted by the small cohort size and require validation in larger, independent patient populations to confirm robust statistical inference.

### 3.7 Exploratory analysis of systemic immune remodeling and the tumor immune microenvironment stratified by KRAS status

**Figure 6:**
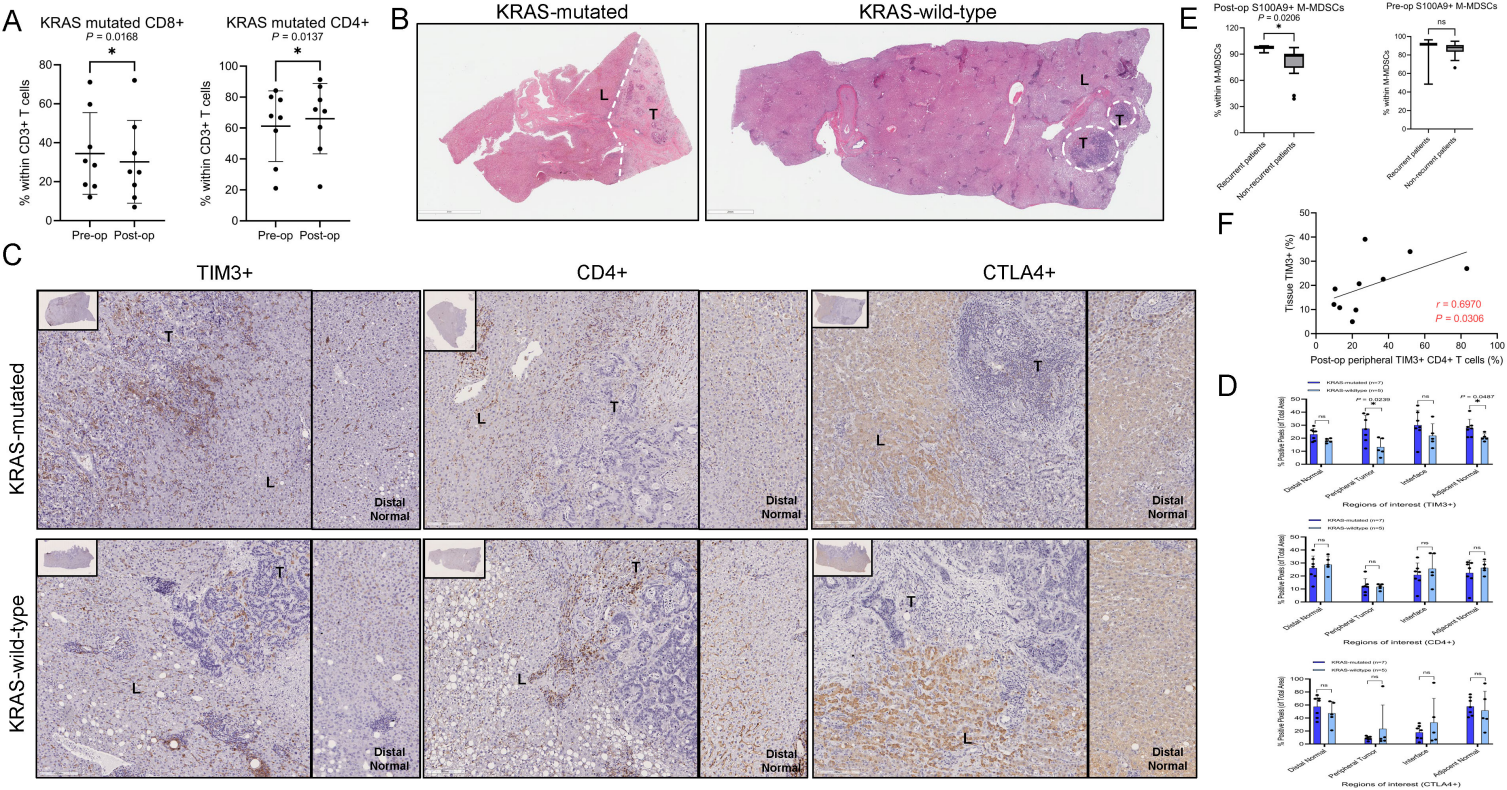
Subgroup analyses of adaptive and innate immune cell subsets under different stratification strategies. **(A)** Frequencies of CD8+ and CD4+ T cells in KRAS-mutated patients (n = 8) before and after liver surgery. **(B)** Frequencies of representative H&E staining of KRAS-mutated and wild-type CRLMs specimens, showing overall tumor morphology and stromal architecture (magnification 4×). **(C)** Immunohistochemical detection of TIM3, CD4, and CTLA4 positive cells in regions of interest of KRAS-mutated (n = 7) and wild-type (n = 5) patients (magnification 40×). **(D)** Quantitative analysis of IHC-positive cells in different tumor regions. **(E)** Frequencies of S100A9+ M-MDSCs in patients stratified by recurrence status (recurrent, n = 3; non-recurrent, n = 14) at preoperative and postoperative time points. Data are presented as mean ± SD or median ± IQR, according to data distribution. **(F)**, Spearman correlation analysis between the percentage of postoperative peripheral TIM3+ CD4+ T cells and CRLM tissue TIM3+ cells (n = 10).

Given the distinct preoperative immune features between KRAS-mutated and wild-type patients, we next assessed how KRAS status influenced postoperative systemic immune remodeling by analyzing paired pre- and postoperative samples within each subgroup. Consistent with our previous observations, CD8+ T-cell frequencies significantly decreased following liver resection, whereas CD4+ T cells markedly increased following liver resection in the KRAS-mutated group (**Figure 6A**). In contrast, these dynamic changes were not observed in the KRAS-wild-type group (**Supplementary Figure 7A**), and baseline levels of CD8+ and CD4+ T cells did not differ between KRAS-mutated and wild-type subgroups (**Supplementary Figure 7B**).

In the tumor immune microenvironment (TIME), H&E staining of resected tumor specimens revealed preserved tumor architecture with variable stromal components across patients (**Figure 6B**). To further characterize the TIME in relation to KRAS status, we performed immunohistochemical and immunofluorescence analyses on surgical tumor tissues from seven KRAS-mutated and five KRAS–wild-type patients. Quantitative IHC assessment demonstrated significantly higher TIM3 expression at the tumor periphery and adjacent liver in KRAS-mutated patients compared with KRAS-wild-type patients. By contrast, CD4 and CTLA4 expression did not differ significantly across four tumor regions between KRAS-mutated and wild-type groups (**Figures 6C, D**). In immunofluorescence analyses (**Supplementary Figures 8A, B**), we observed significantly elevated TIM3 expression at the peripheral tumor region in KRAS-mutated tumor. Moreover, CD68+ TIM3+ double-positive macrophages at the tumor-liver interface of CRLM exhibited an increasing trend in KRAS-mutated tumors compared with KRAS-wild-type tumors (mean percentage: 22.52% vs. 8.84%) (**Supplementary Figure 8C**). These findings suggest that KRAS mutation may contribute to the establishment of a more immunosuppressive myeloid microenvironment in CRLM. Additionally, correlation analysis demonstrated that the percentage of postoperative peripheral TIM3+ CD4+ T cells was positively correlated with tissue TIM3+ cell infiltration (*r* = 0.6970, *P* = 0.0306; **Figure 6F**).

These preliminary findings demonstrate a trend toward a distinct immune phenotype in KRAS-mutated patients, although our current sample size precludes a definitive conclusion regarding a true KRAS-driven divergence in perioperative systemic immune remodeling.

### 3.8 Postoperative S100A9+ M-MDSCs and recurrence

To explore potential links between immune profiles and clinical outcomes, patients were further stratified by recurrence status. Notably, postoperative levels of S100A9+ M-MDSCs were significantly higher in patients who experienced recurrence (p = 0.0206), whereas no differences were observed preoperatively (**Figure 6E**). Given the limited sample size and small number of recurrent cases, this observation should be interpreted with caution and considered exploratory, thereby suggesting that postoperative S100A9+ M-MDSCs may represent a preliminary, recurrence-associated immune feature requiring validation in larger cohorts.

### 3.9 Sensitivity analysis including patients with very early postoperative recurrence

To address potential selection bias and evaluate the robustness of our findings, we performed an exploratory sensitivity analysis including those patients that were excluded due to early postoperative recurrence (defined as recurrence within two months after surgery; n=5). In contrast to the immune remodeling patterns observed in the primary disease-free cohort, these patients did not demonstrate significant postoperative increases in the proportion of circulating CD4+ T cells or CD28+ CD4+ T cells, nor reductions in the proportion of TIM3+ CD4+ or CD8+ T cells (**Supplementary Figures 9A, B**). Notably, a trend towards an increased frequency of TIM3+ CD8+ T cells was observed postoperatively, although this did not reach statistical significance (**Supplementary Figure 9C**). Thus, this was a study design choice focusing on patients with an initially disease-free postoperative course, while acknowledging that very early recurrence cases may represent an important subgroup requiring separate investigation.

Furthermore, elevated postoperative S100A9+ M-MDSCs levels remained associated with recurrence even after inclusion of these early recurrence cases (**Supplementary Figure 9D**). Although limited by the small sample size, these findings suggest that the favorable postoperative systemic immune remodeling observed in the primary cohort may be attenuated or absent in patients with exceptionally early disease progression.

## 4 Discussion

The liver is a common site for metastatic spread, establishing both a local immune-tolerant microenvironment and systemic immune tolerance that promotes the progression of colorectal cancer liver metastasis (11, 35). To our knowledge, this study provides evidence that surgical removal of liver metastases is associated with a marked increase in the proportion of CD4+ T cells among circulating CD3+ T cells and a relative decrease in the proportion of CD8+ T cells. Notably, major CD4+ T-cell lineages implicated in tumor-promoting activity within the tumor microenvironment (Treg and Tfh) (36), did not exhibit significant changes in circulation following liver resection, suggesting that the postoperative expansion of CD4+ compartment arises from other CD4+ population. A recent CRLM mouse model report suggests that liver metastases siphon and eliminate antigen-specific CD8+ T cells from circulation, causing systemic immunosuppression (11). In contrast to that mouse model, no rebound of circulating CD8+ T cells was observed following surgical resection in our study. Instead, the postoperative period was characterized by a significant rise in the frequency of CD4+ T cells, indicating a distinct pattern of systemic immune remodeling in patients. Importantly, we found that systemic CD4+ T cells exhibited a significant increase in CD28 expression postoperatively, whereas CD28 expression on CD8+ T cells remained unchanged. This observation may reflect changes in CD4+ T cell co-stimulatory marker expression, consistent with the established role of CD28 in supporting T cell immunity (37). Together, our data point to an early postoperative shift in systemic immunity that is dominated by quantitative and phenotypic changes in the CD4+ compartment. However, the observed postoperative decline in the frequency of circulating CD8+ T cells warrants a cautious and balanced interpretation, as it does not inherently contradict the concept of systemic immune remodeling. Rather than indicating an impairment of cytotoxic immunity, this peripheral contraction could potentially reflect a dynamic redistribution or trafficking of lymphocytes. It is plausible that the surgical removal of the tumor mass abruptly eliminates the primary source of tumor-derived antigens, thereby altering systemic chemokine gradients and prompting circulating CD8+ T cells to redistribute into secondary lymphoid organs or homing back into peripheral tissues. Alternatively, the transient suppressive impact of surgical stress and wound healing inflammation on certain T-cell subsets cannot be entirely ruled out. Given these physiological possibilities, our findings regarding CD8+ dynamics should be interpreted with prudence, and future studies incorporating functional assays are needed to elucidate whether this peripheral reduction influences long-term antitumor efficacy.

In multiple solid tumors, canonical inhibitory receptors, commonly known as immune checkpoints, including PD-1, CTLA4, TIM3, LAG3 have been detected on CD4+ T cells, mirroring the exhaustion phenotypes typically seen in CD8+ T cells (34, 38). Importantly, the upregulation of immune checkpoints that define T cell exhaustion, particularly TIM3, has been linked to the development of tumor resistance to checkpoint blockade therapies (39). Our study revealed a significant downregulation in the frequency of TIM3+ CD4+ T cells, along with a declining trend in both PD-1+ CD4+ T cells and exhausted PD-1+ TIM3+ double-positive T-cell subsets following surgical resection. Additionally, we observed a similar expression pattern of TIM3 and PD-1 on CD8+ T cells post-surgery. Our data shows that removal of liver metastases is associated with a reduced frequency of T cells expressing immune checkpoint molecules, indicating that the tumor cells induce the upregulation of these molecules, supporting tumor growth and evasion of the immune system. Taken together, our results hypothetically suggest the potential of adoptive CD4+ T cell therapy, in combination with immunotherapy, as a strategy to reinforce systemic immunity and target circulating minimal residual disease (MRD), a major contributor to postoperative recurrence in CRLM. However, these translational interpretations should be considered with caution, as no functional immune assays were performed to evaluate T-cell activity after resection. Accordingly, the clinical relevance of these immune changes in terms of anti-tumor efficacy and immunotherapy response remains to be validated. Future studies should aim to validate the clinical efficacy of adoptive CD4+ T cell therapy and to delineate functionally distinct CD4+ T cell subsets with potent anti-tumor activity, thereby informing optimized production strategies for solid tumor immunotherapy.

Neoadjuvant chemotherapy is commonly employed in patients with advanced CRC, particularly those with liver metastases, to reduce tumor burden and assess tumor chemosensitivity (40). A propensity score-matched retrospective study has suggested that neoadjuvant chemotherapy may impair systemic anti-tumor immune responses in chemotherapy-insensitive breast cancer patients, primarily through a reduction in the proportions of CD4+ T cells and NK cells (41). In contrast, other reports indicate that neoadjuvant chemotherapy can inhibit tumor progression and modulate the tumor microenvironment (TME) by remodeling cancer-associated fibroblasts in rectal cancer (42). In our cohort, neoadjuvant chemotherapy did not significantly alter systemic immune cell subsets at either the preoperative or postoperative time point. However, responders exhibited lower frequencies of circulating M-MDSCs and higher proportions of APCs such as monocytes, suggesting a potentially more favorable systemic immune profile associated with chemotherapy sensitivity.

When analyzing clinical parameters, we observed that the size of the tumor (i.e. tumor burden) was correlated with more B cells in circulation. This would suggest a prolonged immune impact for large masses which needs to be further investigated by screening additional time points. This was not designed in this study but warrant investigation.

KRAS mutations in CRC are well recognized to confer poorer clinical outcomes compared with KRAS-wild-type disease (43). Mechanistically, oncogenic KRAS fosters an immunosuppressive tumor microenvironment through the KRAS–IRF2 (interferon regulatory factor 2) signaling axis, thereby facilitating recruitment of myeloid-derived suppressor cells (MDSCs) and reducing intratumoral T-cell infiltration—particularly CD4+ T cells in primary tumors (44). In our cohort, surprisingly, we observed a marked postoperative increase in the proportion of circulating CD4+ T cells exclusively in patients with KRAS-mutated liver metastases, suggesting that these individuals may experience systemic immune suppression prior to surgery. Concordantly, TIM3 was enriched in tumor tissues, indicative of a suppressive tumor immune microenvironment (TIME). The absence of baseline differences in circulating CD4+ T-cell levels between KRAS-mutated and wild-type groups likely reflects the limited sample size. Together, these findings suggest that postoperative immune remodeling is influenced by multiple factors—including surgical trauma and other immune-regulatory mechanisms—rather than being solely determined by KRAS mutation status. This underscores the interplay between oncogenic signaling and systemic immune dynamics in shaping patient-specific postoperative immunity.

Interestingly, we further observed that patients who experienced recurrence exhibited a higher frequency of S100A9+ M-MDSCs following liver resection. This finding aligns with our earlier work (45), which demonstrated that elevated levels of circulating extracellular vesicle (EV)-associated S100A9, originating from MDSCs, were linked to the poorest prognosis in patients with colorectal cancer liver metastases and early recurrence. While this observation is consistent with prior findings, the limited number of recurrent cases precludes definitive conclusions; therefore, it should be viewed as a preliminary, hypothesis-generating signal that warrants validation in larger, independent cohorts.

Despite the significant findings of this study, certain limitations should be acknowledged. The limited follow-up duration restricted survival analysis, constraining the evaluation of long-term prognostic impacts of the observed immune alterations. Furthermore, the sample size is limited, due to the combination of strict eligibility criteria for confirming disease-free status and the inherent rarity of such cases in clinical settings. Due to this sample size constraint, we were unable to perform multivariable adjustments to control other perioperative confounders (e.g., surgical extent or systemic therapies), which remains a limitation of the current study. In particular, the very small number of recurrent cases (n=3) limits the robustness of subgroup analyses related to recurrence. Despite these constraints, this study uniquely highlights immune dynamics surrounding liver resection, offering valuable perspectives for future research and clinical management.

## 5 Conclusion

Collectively, our data indicate that resection of liver metastases is associated with systemic immune remodeling, hypothetically suggesting a postoperative window of opportunity for immunotherapeutic interventions. Consistent with a prior preclinical study (11) demonstrating that removal of hepatic metastases rendered extrahepatic lesions responsive to systemic immunotherapy, we observed changes in circulating T cell subsets following metastasectomy. The CD4+ T cell profile remodeling warrants further investigation regarding its potential relevance to adoptive cellular therapies or immune checkpoint inhibition. While highly speculative given the current lack of functional assays, further validation of these phenotypes might help elucidate novel strategies to enhance anti-tumor immune responses and improve outcomes in patients with colorectal cancer liver metastases. Prospective, adequately powered clinical trials and long-term translational studies are warranted to further evaluate S100A9+ M-MDSCs and other immune parameters as potential biomarkers associated with recurrence risk and to define optimal perioperative immunotherapeutic strategies. Together, these findings indicate that systemic immune profiling not only captures tumor-induced immune remodeling but also offers potential clinical utility for patient stratification and therapeutic monitoring.

## Data Availability

The raw data supporting the conclusions of this article will be made available by the authors, without undue reservation.
