# Supplementary material for: Systemic immune remodeling following curative (R0) resection of colorectal liver metastases
Source: Front Immunol. 2026 Jul 9;17:1843400. doi: 10.3389/fimmu.2026.1843400 (PMC13391566; doi:10.3389/fimmu.2026.1843400)
Supplement: Supplementary file 2 [file Table2.pdf]

Supplementary Table 2. Patient demographics		
Parameter		All subjects n=17 (%)
Centre		
	MUHC	17 (100%)
Age (years) (mean ± SD)		65.8 ± 13.0
	<65	10 (58.8)
	≥65	7 (41.2)
Sex		
	Male	11 (64.7)
	Female	6 (35.3)
BMI (kg/m2) (mean ± SD)		28.4 ± 6.0
Follow-up time (month) (mean ± SD)		9.0 ± 4.86
Primary tumor location		
	Right	7 (41.2)
	Left	2 (11.8)
	Sigmoid	5 (29.4)
	Rectum	3 (17.6)
Primary tumor T stage		
	T0	0 (0)
	T1	0 (0)
	T2	1 (5.9)
	T3	7 (41.2)
	T4	9 (52.9)
Primary tumor N stage		
	N0	6 (35.3)
	N1	4 (23.5)
	N2	7 (41.2)
KRAS status		
	mt	10 (58.8)
	wt	7 (41.2)
BRAF status		
	mt	1 (5.9)
	wt	16 (94.1)
PIK3CA status		
	mt	2 (11.8)
	wt	15 (88.2)
ERBB2 status		
	mt	1 (5.9)
	wt	16 (94.1)

Mismatch repair		
	Proficient	16 (94.1)
	Deficient	1 (5.9)
Timing of metastases		
	Metachronous	3 (17.6)
	Synchronous	14 (82.4)
Adjuvant CTX following CRC resection		
	Yes	10 (58.8)
	No	7 (41.2)
Times of liver resection		
	First-time metastasis	13 (76.5)
	Recurrent metastasis	4 (23.5)
Neoadjuvant CTX before liver resection		
	Yes	11 (64.7)
	No	6 (35.3)
Number of metastases (median [IQR])		1.0 [1.0, 3.0]
	1	10 (58.8)
	>1	7 (41.2)
Size of largest metastases (mean ± SD)		3.8 ± 2.2
	≤5 cm	14 (82.4)
	>5 cm	3 (17.6)
Distribution of metastases		
	Bilobar	3 (17.6)
	Unilobar	14 (82.4)
Preoperative CEA before liver resection (ng/ml) (median [IQR])		7.9 [4.3, 41.6]
	<10ng/ml	11 (64.7)
	≥10ng/ml	5 (29.4)
	Missing	1 (5.9)
