# Supplementary figures and images for: Systemic immune remodeling following curative (R0) resection of colorectal liver metastases
Source: Front Immunol. 2026 Jul 9;17:1843400. doi: 10.3389/fimmu.2026.1843400 (PMC13391566; doi:10.3389/fimmu.2026.1843400)

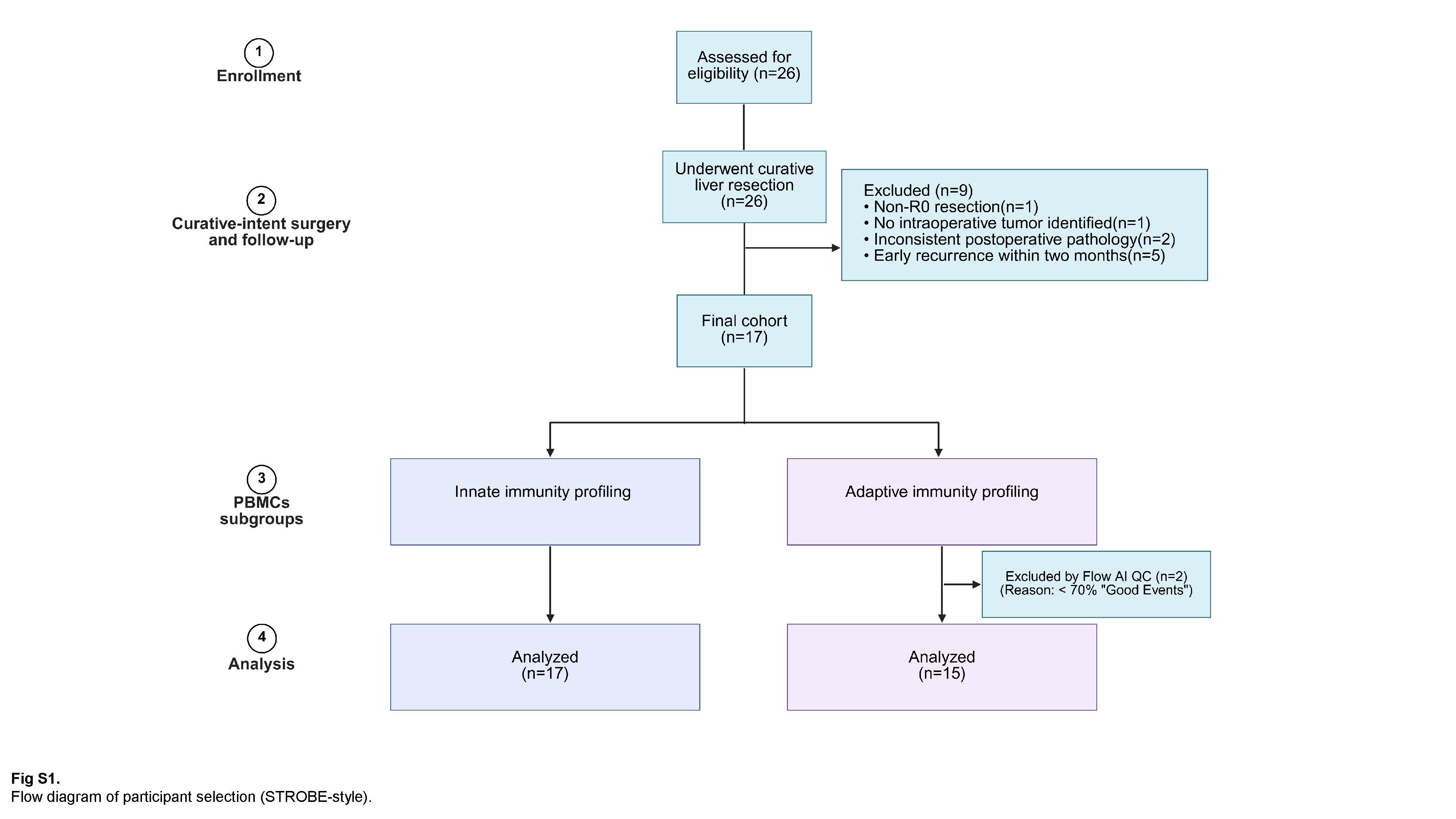

Supplement: Supplementary file 4 [file Image1.tiff]

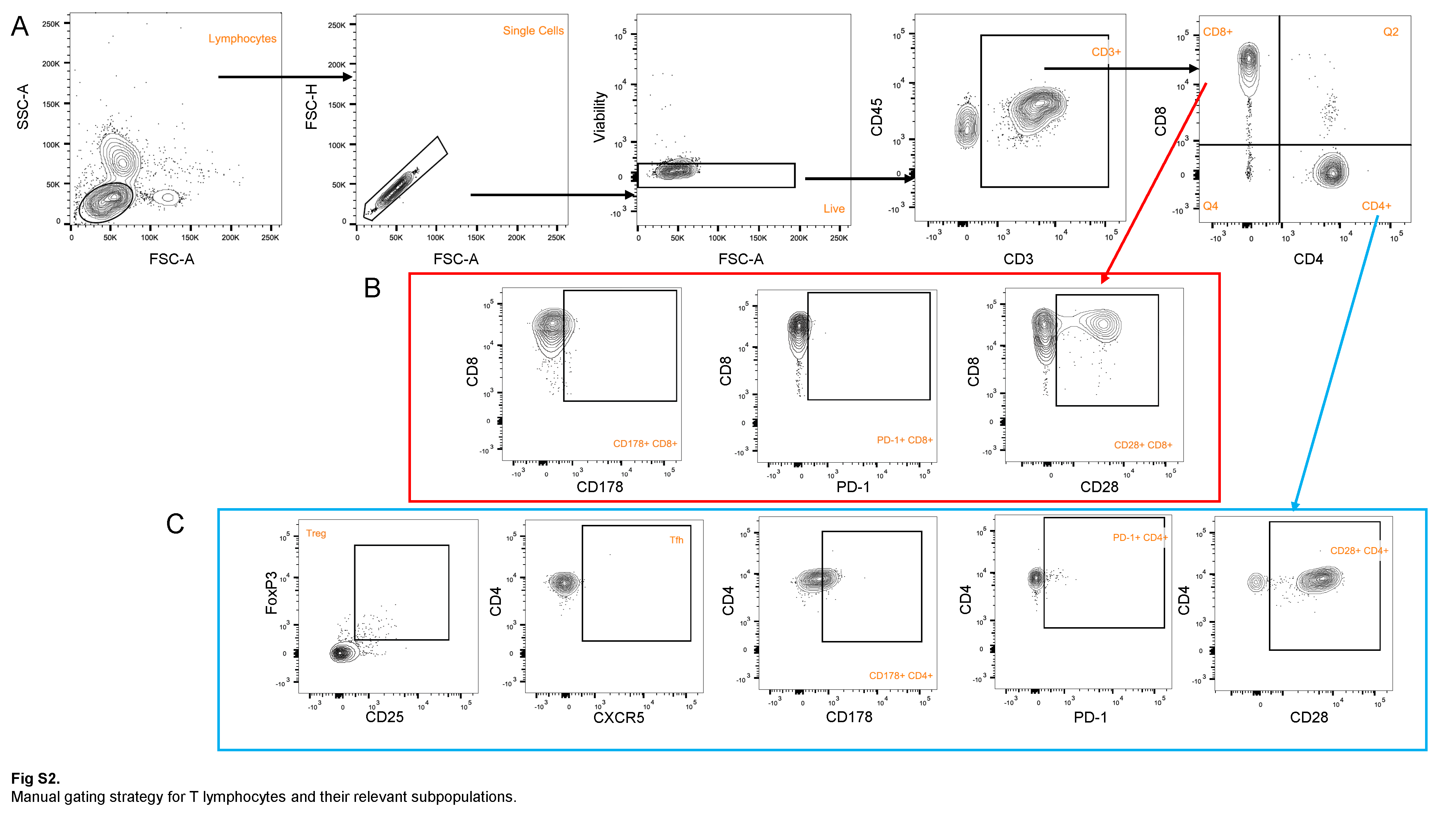

Supplement: Supplementary file 5 [file Image2.tiff]

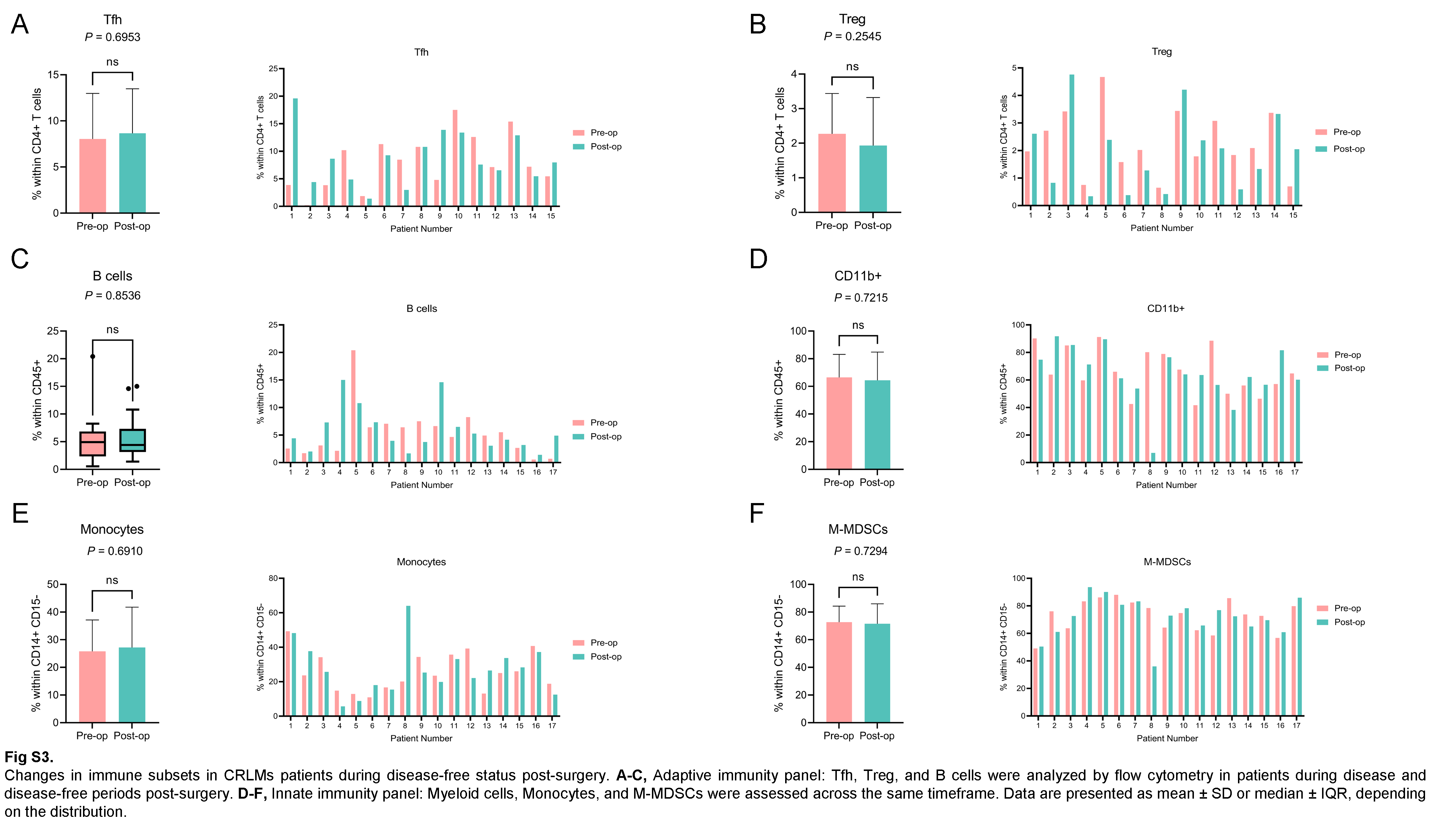

Supplement: Supplementary file 6 [file Image3.tiff]

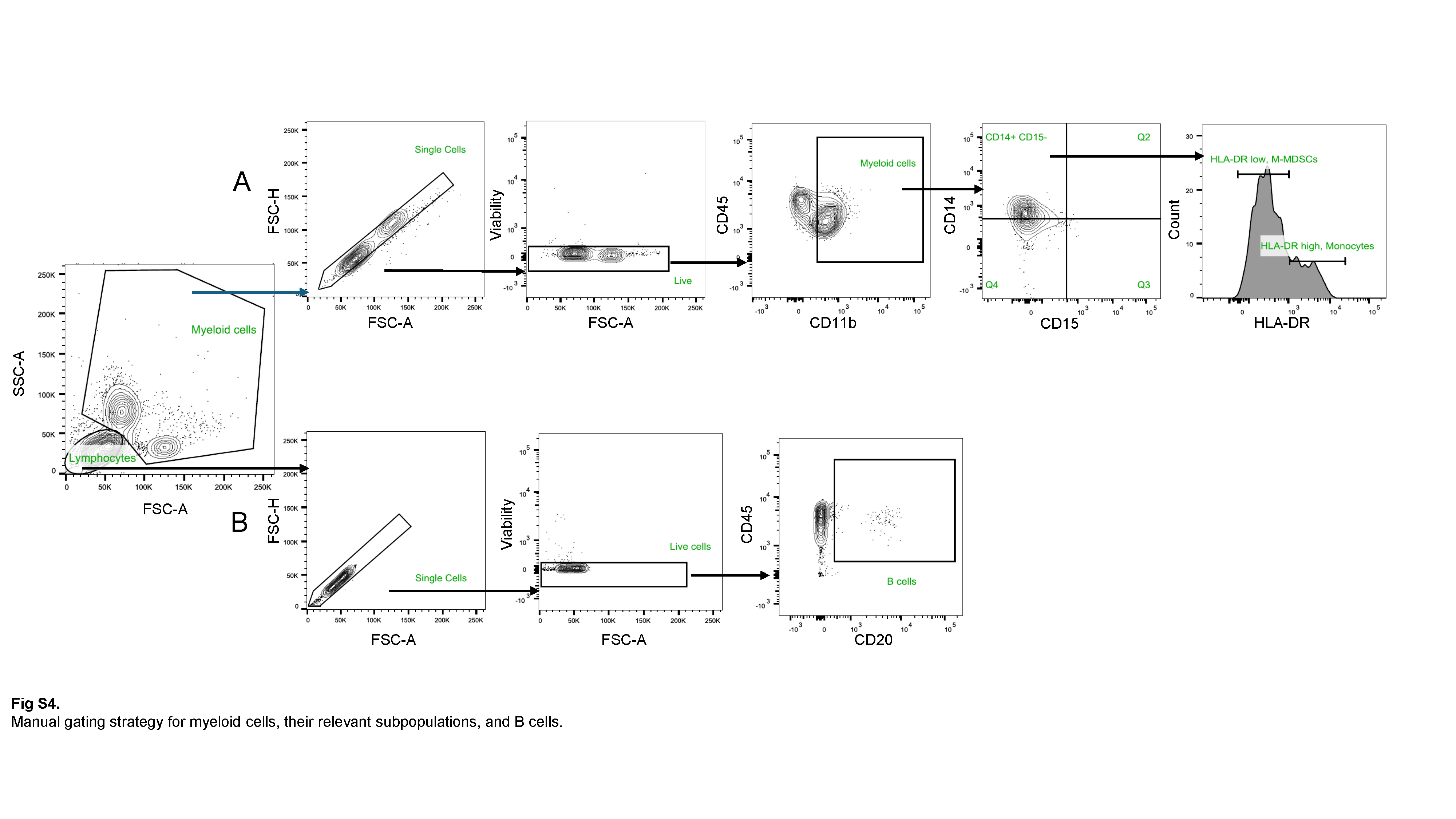

Supplement: Supplementary file 7 [file Image4.tiff]

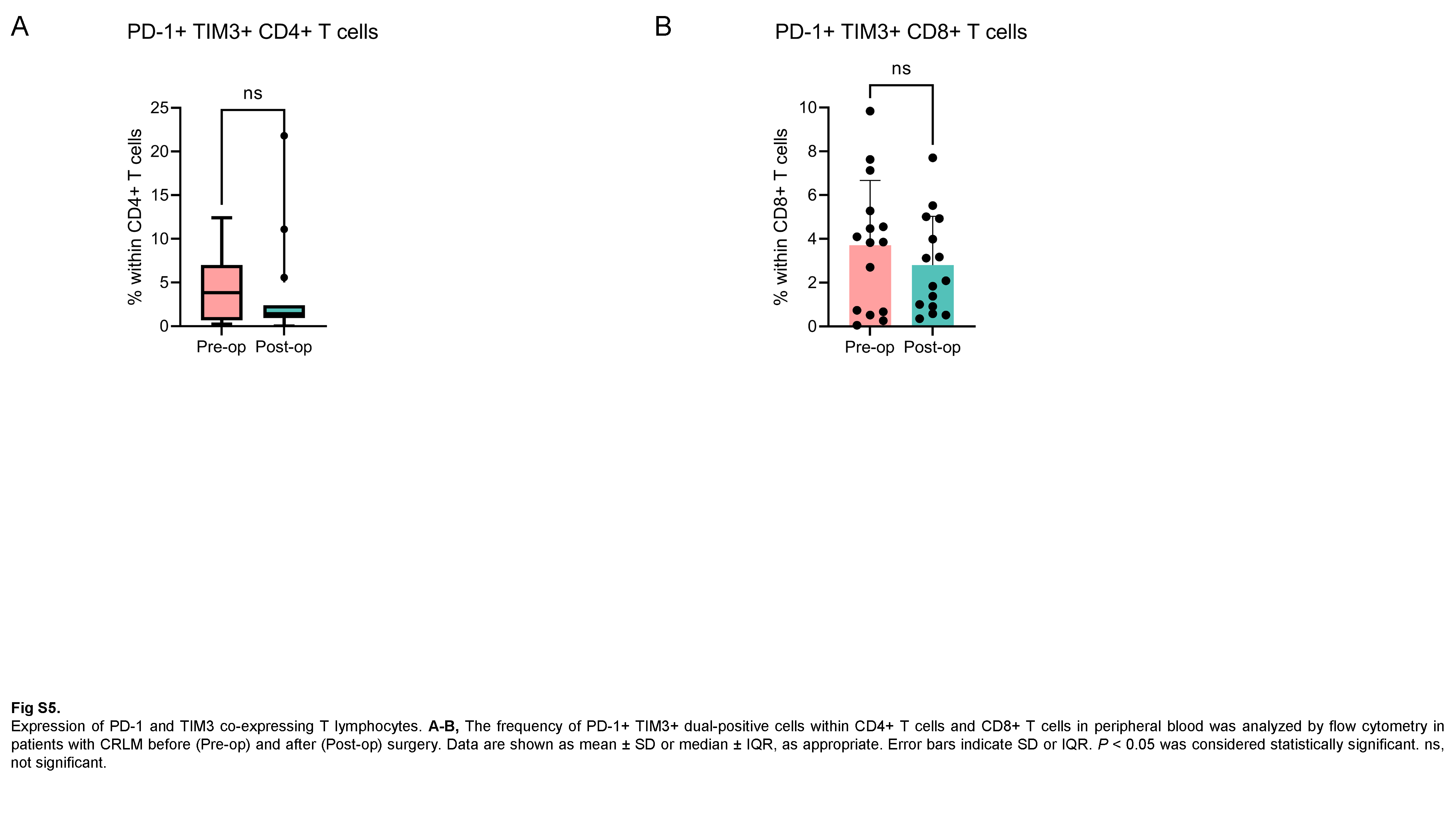

Supplement: Supplementary file 8 [file Image5.tiff]

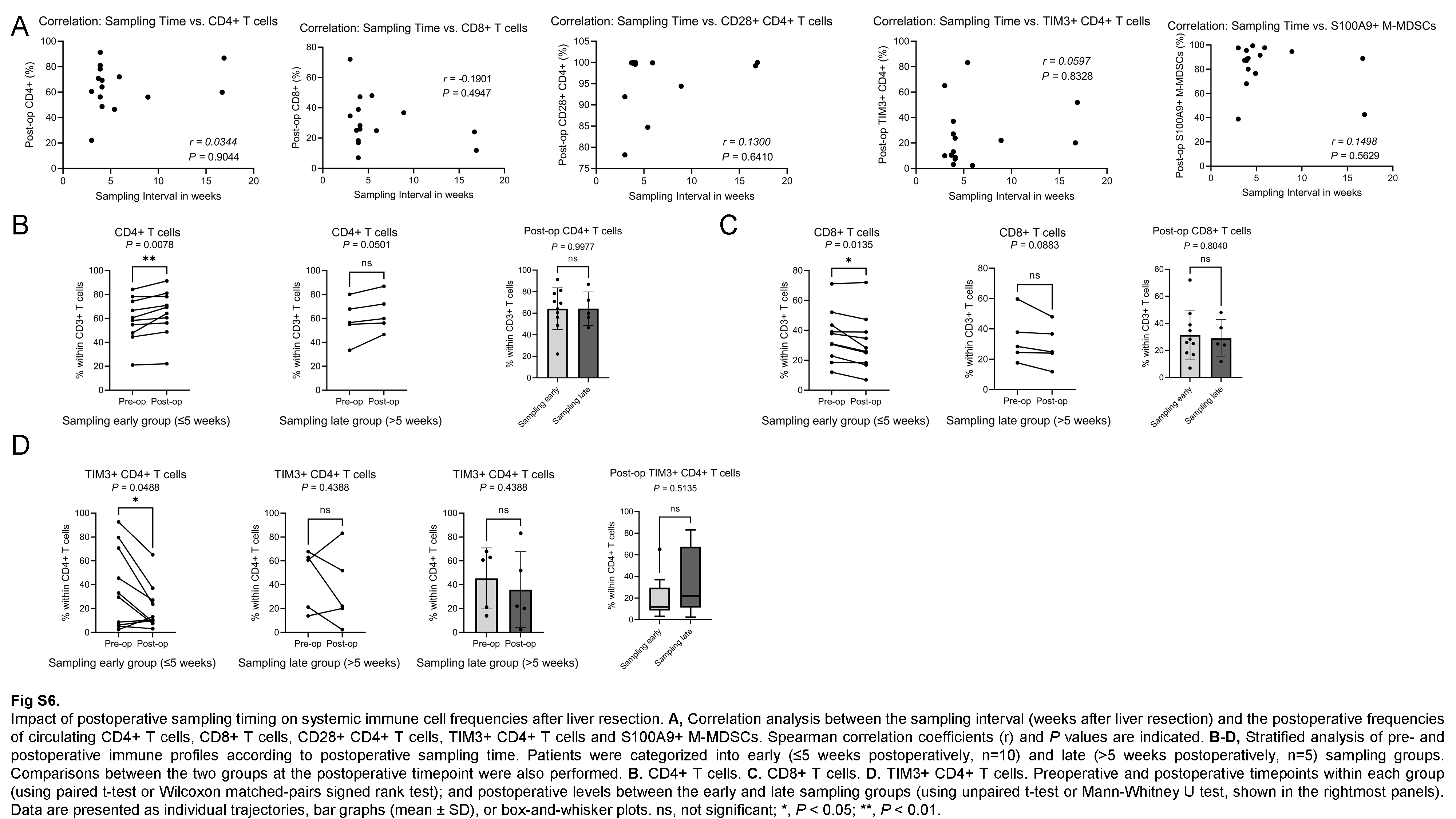

Supplement: Supplementary file 9 [file Image6.tiff]

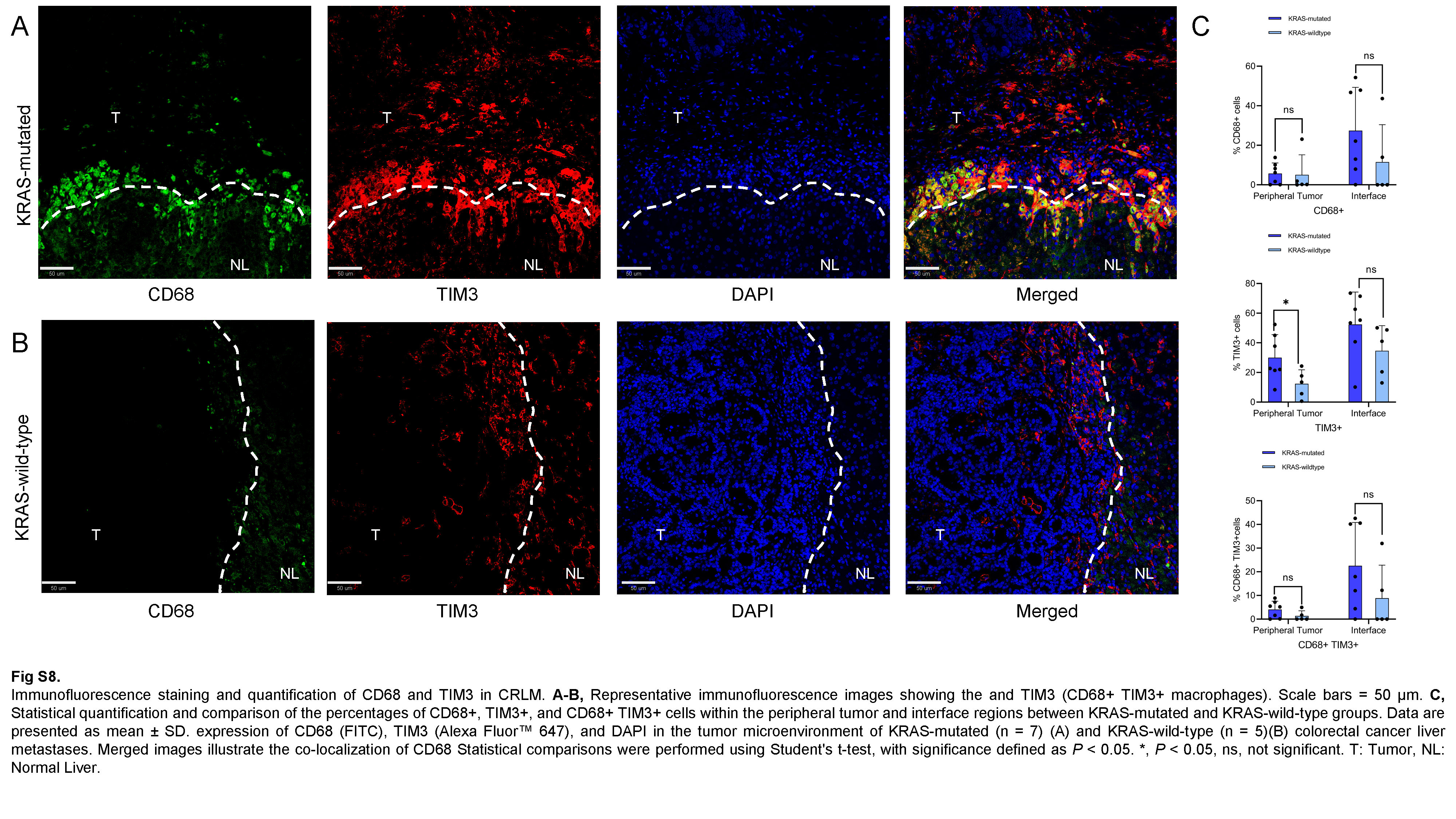

Supplement: Supplementary file 11 [file Image8.tiff]

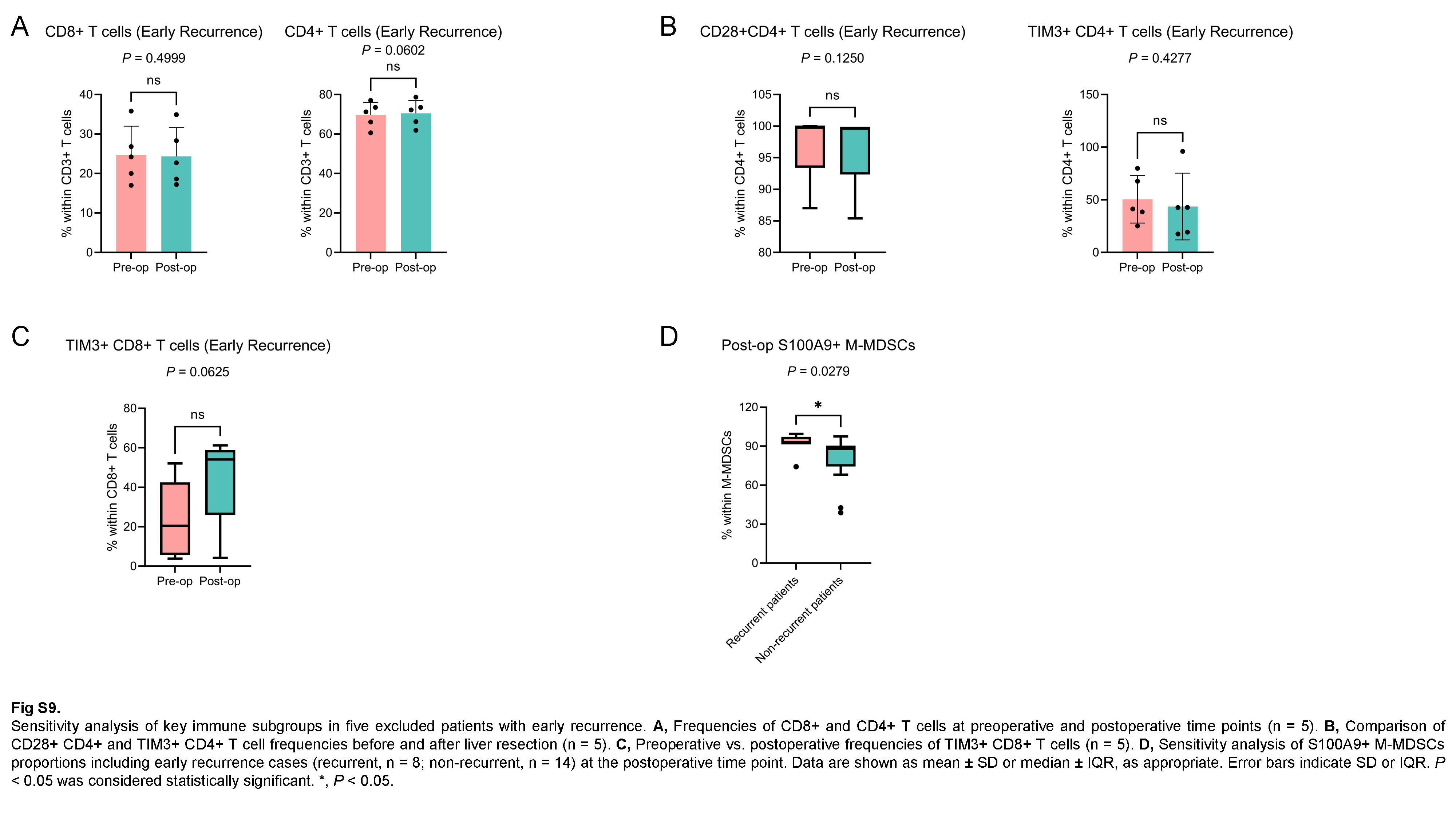

Supplement: Supplementary file 12 [file Image9.tiff]
